# Ischemia-Triggered Glutamate Excitotoxicity From the Perspective of Glial Cells

**DOI:** 10.3389/fncel.2020.00051

**Published:** 2020-03-19

**Authors:** Denisa Belov Kirdajova, Jan Kriska, Jana Tureckova, Miroslava Anderova

**Affiliations:** ^1^Department of Cellular Neurophysiology, Institute of Experimental Medicine, Academy of Sciences of the Czech Republic (ASCR), Prague, Czechia; ^2^Second Faculty of Medicine, Charles University, Prague, Czechia

**Keywords:** ischemic pathway, glutamate excitotoxicity, glutamate uptake/release, cell death, astrocytes, oligodendrocytes, NG2 glia, glutamate receptors and transporters

## Abstract

A plethora of neurological disorders shares a final common deadly pathway known as excitotoxicity. Among these disorders, ischemic injury is a prominent cause of death and disability worldwide. Brain ischemia stems from cardiac arrest or stroke, both responsible for insufficient blood supply to the brain parenchyma. Glucose and oxygen deficiency disrupts oxidative phosphorylation, which results in energy depletion and ionic imbalance, followed by cell membrane depolarization, calcium (Ca^2+^) overload, and extracellular accumulation of excitatory amino acid glutamate. If tight physiological regulation fails to clear the surplus of this neurotransmitter, subsequent prolonged activation of glutamate receptors forms a vicious circle between elevated concentrations of intracellular Ca^2+^ ions and aberrant glutamate release, aggravating the effect of this ischemic pathway. The activation of downstream Ca^2+^-dependent enzymes has a catastrophic impact on nervous tissue leading to cell death, accompanied by the formation of free radicals, edema, and inflammation. After decades of “neuron-centric” approaches, recent research has also finally shed some light on the role of glial cells in neurological diseases. It is becoming more and more evident that neurons and glia depend on each other. Neuronal cells, astrocytes, microglia, NG2 glia, and oligodendrocytes all have their roles in what is known as glutamate excitotoxicity. However, who is the main contributor to the ischemic pathway, and who is the unsuspecting victim? In this review article, we summarize the so-far-revealed roles of cells in the central nervous system, with particular attention to glial cells in ischemia-induced glutamate excitotoxicity, its origins, and consequences.

## Introduction

Glutamate excitotoxicity is a cell death mechanism triggered by excessive glutamate release from neurons as well as glial cells. It was described almost 50 years ago as “a certain kind of regionally specific neuropathology” in the hypothalamus of infant mice (Olney, 1971). Glutamate excitotoxicity may develop during numerous events; as a secondary injury after traumatic injury (Park et al., 2008), during various brain pathologies, such as Alzheimer’s (Tannenberg et al., 2004), Parkinson’s (Verma et al., 2018), or Huntington’s disease (Warby et al., 2008; Girling et al., 2018) or during ischemia, which is the main topic of this review article. Despite the different etiology of brain damage, strikingly similar consequent events are triggered (Bramlett and Dietrich, 2004; Amani et al., 2019), which could possibly lead to the development of a common treatment for a number of disorders in the central nervous system (CNS). Under physiological conditions, neuronal cells heavily depend on glia. Astrocytes, the most abundant glial cell type in the brain, have many functions, as they scavenge neurotransmitters, shuttle metabolites and maintain homeostasis in the CNS. However, during ischemia, they fail to carry out these functions and become a threat to the adjacent neurons. Thus, glial cells can act as defenders of the CNS as well as initiators and propagators of ischemic injury (Takano et al., 2009).

## Ischemia

Ischemia is a medical condition in which poor blood flow to the tissue causes oxygen and glucose deprivation (OGD), both leading to cell death (Puig et al., 2018). A fall in the number of neurons, followed by a decrease in the counts of glia is apparent especially in the brain parenchyma, due to its high demand for aerobic metabolism. The adult human brain represents a relatively small proportion of the body weight (~2%), but it accounts for more than 20% of the whole body energy budget (Doyle et al., 2008). Normal cerebral blood flow (CBF) is approximately 50 ml/100 g/min and ischemia occurs when the CBF drops under 40% of its physiological values (Baron, 2001; Heiss et al., 2004). When below a CBF of 10 ml/100 g/min, rapid irreversible damage to neurons and oligodendrocytes ensues (Matsumoto et al., 1990; Baltan, 2016). The typical symptoms of brain ischemia range from mild to severe and may encompass dizziness, impairments in vision and body movement, difficulty speaking, coordination issues, and unconsciousness (Nadarajan et al., 2014). Ischemic stroke is one of the leading causes of mortality worldwide, surpassed only by cancer and cardiovascular diseases (Doyle et al., 2008). It is estimated that the lifetime risk of cerebral stroke is at 1 in 6 middle-aged (55–75-year-old) adults (Seshadri et al., 2006) and the death rates are lower in more developed countries, with ~15% mortality (Marini et al., 1999). According to the location and extent of the injury, ischemia can be divided into two subtypes: focal and global. Focal cerebral ischemia (FCI) is caused by the occlusion of specific arteries of the brain, while global cerebral ischemia (GCI) stems from an overall decrease in blood flow (Yao et al., 2018).

## Focal Cerebral Ischemia

**Figure 1:**
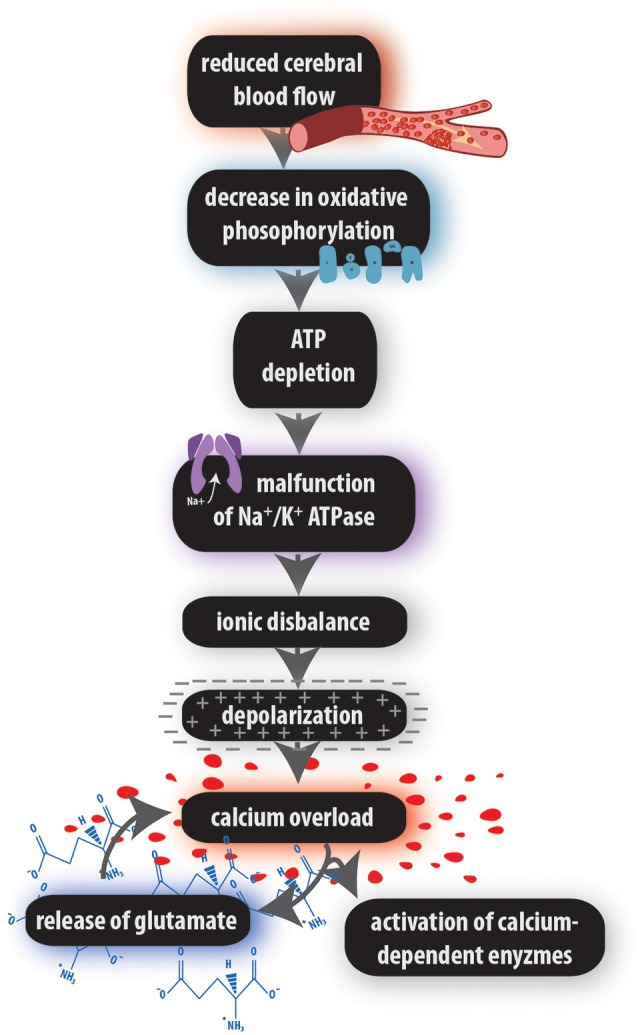
Ischemic cascade. The relay of extracellular and intracellular processes leading to pathogenic states and eventually to cell death.

As the name suggests, FCI (or stroke) is confined to a locally defined region of the brain. and is caused by thrombosis, or embolism (VanGilder et al., 2012). In the affected area of nervous tissue close to the occluded vessel, two distinct zones can be distinguished: infarction core (a zone of severe ischemia) and penumbra (a zone of moderate ischemia; Rossi et al., 2007), and the size of both of these zones is almost equivalent (Ginsberg, 2003); however, as the ischemic injury progresses, the penumbra becomes “devoured” by the ischemic core (Zhao et al., 1997). The infarction core is characterized by a lack of adenosine triphosphate (ATP), pathological concentrations of ions, high concentrations of glutamate and tissue acidosis. Cell death occurs within the first minutes after the onset of ischemic injury (Wetterling et al., 2016), partially due to the inability of compromised astrocytes to deliver nutrients to neurons (Chisholm and Sohrabji, 2016). Nevertheless, it is necessary to mention that in the ischemic core, also surviving and even newly formed viable neurons have been detected (Zhang et al., 2017). On the contrary, there is a residual blood flow in the penumbra due to the presence of collateral arteries (Harukuni and Bhardwaj, 2006; Jung et al., 2017), with only lowered concentrations of ATP, to some extent maintained ionic concentrations, recurrent episodes of cortical spreading depression (or peri-infarct depolarization; Hinzman et al., 2015; Oliveira-Ferreira et al., 2019), and apoptosis as typical cellular death (Rossi et al., 2007; Doyle et al., 2008; Figure 1). Moreover, glial elements, astrocytes and NG2 glia, in this area form the so-called glial scar that prevents detrimental compounds from entering the spared nervous tissue (Adams and Gallo, 2018), while NG2 cells also display a multipotent differentiation potential (Honsa et al., 2012). Despite the substantial cessation of the CBF in the penumbra, the levels of glucose metabolism remain constant due to elevated glucose utilization; however, a normal rate of glucose production is maintained for only ~1 h from the onset of FCI (Belayev et al., 1997).

## Global Cerebral Ischemia

GCI results from transient cardiac arrest causing a systemic and complete cessation of blood flow in the whole parenchyma of the brain (Harukuni and Bhardwaj, 2006), which then diminishes the number of neuronal and glial cells and reduces the integrity of the blood-brain barrier (BBB; Sanganalmath et al., 2017). This type of ischemic injury is characterized by delayed neuronal death (Guo et al., 2019) in specific brain regions of the brain (Kirino and Sano, 1984; Anderova et al., 2011) and by the proliferation and activation of glial cells (Anderova et al., 2011). Moreover, GCI increases the expression of calcium-permeable ion channels in astrocytes, which may influence the output of the ischemic insult (Butenko et al., 2012). The events occurring during GCI are very similar to those found in the core of FCI. While the restoration of CBF is essential for the protection of spared brain tissue (Durán-Laforet et al., 2019), reperfusion may also bring more Ca^2+^, causing further damage (Unal-Cevik et al., 2004; Harukuni and Bhardwaj, 2006; Kauppinen and Swanson, 2007). The disruption of the BBB, caused partly by astrocyte dysfunction, may also complicate the situation (Kahles et al., 2007), causing vasogenic edema and extensive compression of the brain (Thompson and Ronaldson, 2014).

## Phases of Ischemia

Ischemic injury may be manifested to various extents, depending on the duration of blood flow cessation (Heiss et al., 2004). Mild ischemia (CBF greater than 30 ml/100 g/min) does not normally cause cell death during the first 6 h after injury, moderate ischemia (CBF between 12 and 30 ml/100 g/min) reaches the threshold of viability in 3 h, and severe ischemia (CBF lower than 12 ml/100 g/min) causes cell death in less than an hour (Sakoh et al., 2000). As ischemia progresses, it can be further divided into three phases according to the events occurring in the affected tissue: acute (or initial), subacute (intermediate), and chronic (late) phase (Andreeva et al., 2014). In the acute ischemic phase, the tissue is affected by a sequence of molecular and cellular events due to a decrease in the CBF. This sequence of events is called the ischemic pathway, which begins with energy depletion and glutamate excitotoxicity, and ends in cell death (Lai et al., 2014; Cuartero et al., 2016; Figure 1). According to the changes in membrane potential, the most vulnerable cells to 30-min OGD are neurons, followed by astrocytes, while NG2 glia showed no significant pathological alterations (Du et al., 2018). Moreover, microglial cells are least vulnerable to ischemia because they express glutamate receptors only after the onset of pathological conditions when they become reactive (Gottlieb and Matute, 1997). During the subacute phase (24–72 h after the stroke onset), vasogenic edema begins (Wetterling et al., 2016). However, weeks after the onset of ischemia, the chronic phase leads to additional tissue damage and may be the result of delayed neurodegeneration triggered by oxidative stress and immune activation. All three phases of ischemic injury are characterized by specific patterns of gene expression (Andreeva et al., 2014), and whereas neuronal necrosis occurs early in the ischemic pathway and negatively affects neighboring neurons and glia, delayed neuronal death emerges several days or even weeks after primary damage due to apoptosis (Du et al., 1996; Zhang J. et al., 2012).

## Ischemic Pathway

**Figure 2:**
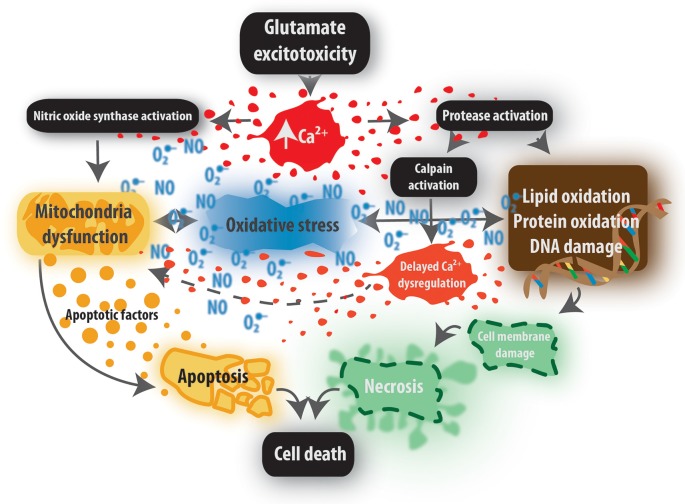
Excitotoxic events caused by aberrant Ca^2+^ levels. High concentrations of intracellular Ca^2+^ lead to the activation of Ca^2+^-dependent enzymes, such as proteases and nitric oxide synthase (NOS). This results in the dysfunction of mitochondria, oxidative stress, and oxidation of essential macromolecules, all contributing to apoptosis or necrosis.

Ischemic injury is usually studied in the adult mammalian brain at the cellular as well as the molecular level (Rossi et al., 2007). Morphological changes, occurring during the activation of microglia and astrocytes, or higher proliferation of NG2 glia, microglia and astrocytes are all hallmarks of ischemia (Davalos et al., 2005; Pforte et al., 2005; Burns et al., 2009; Anderova et al., 2011). Another set of pathogenic events, the aforementioned ischemic pathway (Figure 1), takes place in the nervous tissue affected by ischemia (Amantea et al., 2018). Such a series of biochemical processes is a typical consequence of cardiac arrest or stroke. From minutes up to days after ischemic injury, neurons and glia in the affected area die due to the presence of detrimental chemical compounds activated over time. This mostly linear relay of cellular events starts with a low supply of oxygen and glucose, while oxygen insufficiency completely disrupts mitochondrial oxidative phosphorylation (Erecińska and Silver, 2001). As most of the energy formed in the brain is due to glucose oxidation to carbon dioxide and water (Rama and García, 2016), its absence causes ATP depletion within only a few minutes, which is the result of no long-term energy stores in the brain (Doyle et al., 2008). The lack of ATP hampers the physiological function of ion pumps and, as the cells fail to maintain electrochemical gradients, it results in depolarization due to the aberrant influx of sodium ions (Na^+^) and Ca^2+^ into the cell, accompanied by potassium ion (K^+^) efflux. The elevated extracellular concentration of K^+^ causes the opening of L-type voltage-gated Ca^2+^ channels (Luoma et al., 2011). Ion pumps and exchangers are unable to keep up with the increasing concentrations of Ca^2+^, which is not pumped out of the cell, and high Ca^2+^ levels trigger the release of glutamate, a major contributor to ischemia-induced excitotoxicity in neurons and glial cells (Papazian et al., 2018; Verma et al., 2018; Figure 1). This excitatory amino acid acts as a neurotransmitter and binds to glutamate receptors, of which opening leads to additional Ca^2+^ entry into the lumen of the cell. A sustained activation of Ca^2+^-permeable channels due to an impaired glutamate uptake by astrocytes (Mori et al., 2004) leads to a pathological increase in intracellular Ca^2+^ ([Ca^2+^]_i_) and overexcites the cells which in turn release harmful substances, such as reactive oxygen species (ROS; Sattler et al., 1999) or ATPases, endonucleases, proteases, and phospholipases that belong to a group of Ca^2+^-dependent degradative enzymes. Once the cellular membrane is disrupted by phospholipases, more detrimental chemicals enter the cell, eventually causing the release of apoptotic signals from mitochondria and triggering the caspase-dependent cascade, which leads to the death of cells (Rebai and Amri, 2018; Figure 2). Moreover, another type of detrimental signals spreads from dying cells to viable glia and neurons. The so-called peri-infarct depolarizations are probably triggered by neuronal and glial release of glutamate, which increases inward currents and thus depolarizes cells (Hossmann, 1996; Somjen, 2001; Hartings et al., 2017), and occur in the surroundings of the lesions caused by FCI, beginning in the ischemic core and propagating to the spared regions of nervous tissue (Hossmann, 1996). These recurrent waves of increased concentrations of K^+^ and astrocytic Ca^2+^ cause cytotoxic edema (Hartings et al., 2017) and may potentially result in terminal depolarizations and falls in perfusion, causing secondary expansion of the infarct core at the expense of the penumbral region (Strong et al., 2007; Hinzman et al., 2015; Rakers and Petzold, 2017).

## Glutamate

As already mentioned, glutamate is the driving force of ischemia-induced excitotoxicity. It is the most abundant free amino acid in the CNS, acting as an excitatory neurotransmitter (Pinky et al., 2018). Additionally, under physiological conditions, it plays an important role in learning and memory (Marmiroli and Cavaletti, 2012) and glutamate released from astrocytes synchronizes the activity of hippocampal neurons (Angulo et al., 2004). A large proportion of glutamate in the body comes from the diet; however, it is unable to cross the BBB and must be generated by the resident cells in the CNS. For this reason, this compound is synthesized from its precursor alpha-ketoglutarate, a key molecule in the Krebs cycle (Mark et al., 2001; Rowley et al., 2012). Glutamate is also recycled from glutamine, produced by glial cells in the glutamate-glutamine cycle (Diaz-Ruiz et al., 2016; Hu et al., 2018). In normal synaptic communication, neuronal cells release glutamate from the presynaptic terminals of the axon into the synaptic cleft. The quanta of glutamate accumulated in the extracellular space (ECS) activate glutamate receptors on the surface of postsynaptic terminals (Rowley et al., 2012). Nevertheless, the extracellular levels of glutamate require a tight physiological regulation in order to maintain optimal neuronal excitation and, at the same time, to restrain its excitotoxic effect. The maintenance of glutamate homeostasis largely relies on the activity of astrocytic transporters that under physiological conditions clear glutamate from the synaptic cleft (Bylicky et al., 2018). However, in pathological conditions such as ischemia/reperfusion injury (Volterra et al., 1994; Harvey et al., 2011; Zhang et al., 2019), or during aging (Lewis et al., 2012), the activity of glial glutamate transporters ceases and the abundance of glutamate may damage the surrounding nervous tissue and hamper normal brain functions. Moreover, proinflammatory cytokine tumor necrosis factor α (TNFα) increases the astrocytic expression of glutaminase, which converts glutamine back to glutamate. Since astrocytes are the principal glutamine factory in the CNS, the effect of TNFα counteracts their main mission and serves as a mediator in excitotoxicity-promoting events during inflammation (Milewski et al., 2019). The dysfunction of glutamatergic neurotransmission results in neuronal excitotoxicity, meaning that glutamate excites cells to their death.

## Glutamate Receptors

The release of glutamate is followed by the activation of its postsynaptic receptors. These receptors are of two kinds: ionotropic or metabotropic (mGluRs). Ionotropic, or ligand-gated, receptors are divided into two groups: N-methyl-D-aspartate (NMDA) receptors and non-NMDA receptors, and further classified into three classes, depending on the specific agonist (Atoji and Sarkar, 2019) that binds to the particular receptor: NMDA receptors, α-amino-3-hydroxy-5-methyl-4-isoxazolepropionic acid (AMPA) receptors, and kainate receptors. The excitotoxic injury is mediated mainly *via* glutamate receptors of the NMDA class (Gupta et al., 2013; Girling et al., 2018). Metabotropic receptors are coupled to heterotrimeric guanine nucleotide-binding (G) proteins that relay the signal to its effector channels or intracellular enzymes. These receptors are also divided into three categories, depending on the G proteins they utilize; group I is excitatory (Feng et al., 2019), while groups II and III are inhibitory (Price et al., 2005; Blackshaw et al., 2011). Group-I receptors signal through protein kinase C and phospholipase C, while the latter produces inositol triphosphate. This molecule binds to receptors located on the endoplasmic reticulum, resulting in the Ca^2+^ release into the lumen of the cell (Ribeiro et al., 2010). The inhibitory mGluRs influence adenylyl cyclase that converts ATP to its cyclic form, 3′,5′-cyclic adenosine monophosphate (cAMP), which normally activates protein kinase A (Pin and Duvoisin, 1995). Ionotropic receptors form an ion channel pore and, after the ligand binds to their extracellular domain, the ion channel opens and thus allows the influx of positively charged ions (Na^+^, Ca^2+^). This causes depolarization of the cell membrane, action potential progression, and the release of neurotransmitters from the presynaptic terminals (Mark et al., 2001). Under normal conditions, NMDA receptors are blocked by Mg^2+^ ions. These ions are expelled only after depolarization of the cell, which is achieved by the activation of the non-NMDA receptors that do not possess the Mg^2+^ block. After the ligand binds to its non-NMDA receptor, the channel opens immediately, allowing positive ions (mainly Na^+^) to flow into the cell. Once the Mg^2+^ block is removed from the NMDA receptor, glutamate is able to open the channel and large quantities of Ca^2+^ flow into the cell (Dzamba et al., 2013). Ionotropic receptors of the NMDA type have also been identified on the membranes of astrocytes and oligodendrocytes. Interestingly, these receptors are devoid of Mg^2+^ block and can be thus activated without antecedent depolarization (Salter and Fern, 2005; Lalo et al., 2006). Moreover, glial NMDARs contain GluN3A receptor subunit, which lowers Ca^2+^ permeability (Burzomato et al., 2010; Palygin et al., 2011); however, their permeability to Na^+^ is substantial (Pachernegg et al., 2012) and causes swelling of glial cells, which may aggravate ongoing excitotoxicity during ischemia. Glial cells also possess non-NMDA ionotropic glutamate receptors that were found mainly in oligodendrocytes and astrocytes (Matute et al., 2002). AMPA receptors are composed of 4 subunits, of which the GluR2 subunit determines the Ca^2+^ permeability (Park et al., 2008). Interestingly, TNFα, present at the site of injury (Crespo et al., 2007), increases the synaptic levels of GluR2-lacking receptors and therefore exacerbates the excitotoxic damage (Stellwagen et al., 2005). Moreover, dysfunctional signaling *via* group I mGluRs is thought to lead to defective internalization of GluR2-containing AMPA receptors, which may also influence the permeability of the cellular membrane to Ca^2+^ (Feng et al., 2019). Hyperactivation of glutamate receptors, caused by the surplus of glutamate in the ECS, leads to a massive Ca^2+^ influx. If the energy supply is sufficient, ion pumps take care of the ion equilibrium in the cells and remove some of the positive ions after they have entered the cell (Piccolini et al., 2013). However, if the energy in the cell is low, the ion pumps do not work properly, which leads to a significant increase in the [Ca^2+^]_i_ (Kumagai et al., 2019). Such [Ca^2+^]_i_ increase results in the activation of protein kinases and other downstream Ca^2+^-dependent enzymes that destroy important molecules and disintegrate the cell membrane, causing further Ca^2+^ influx to the cells, release of free radicals from damaged mitochondria, and subsequent cell death (Chan, 2001; Kumagai et al., 2019; Figure 2). Additionally, after glutamate exposure, the concentration of the neurotransmitter ATP in the ECS increases, aggravating the NMDA receptor-mediated cell death (Simões et al., 2018). However, ATP also acts as a modulator, since glutamate released during neuronal activity can activate non-NMDA receptors on astrocytes, which triggers the release of ATP (Zhang et al., 2003). This molecule is released from astrocytes predominantly through connexin 43 hemichannels (Cx43; Kang et al., 2008) and suppresses glutamatergic synapses through presynaptic P2Y receptors (Zhang et al., 2003) or synaptic P2X receptors (Lalo et al., 2016). Here, astrocytes and astrocytic ATP perform as saviors of the CNS, while a similar function has been attributed also to adenosine, which is a product of ATP, released also by glial cells (Zhang et al., 2003). Neurotransmitter receptors for ATP and glutamate are necessary also for oligodendrocyte differentiation and myelination; however, their overactivation during ischemia results in Ca^2+^ overload and mitochondrial dysfunction, which leads to white matter injury. In the view of the fact that white matter is more susceptible to ischemia-induced excitotoxicity (Doyle et al., 2018), especially in striatal white matter of adult individuals (Ahrendsen et al., 2016), oligodendrocytes and NG2 glia should be considered as primary therapeutic targets under hypoxic conditions (Mifsud et al., 2014; Dai et al., 2020).

## Glutamate Transporters

Glutamate uptake *via* EAATs serves as a major mechanism for regulating the extracellular concentration of glutamate and preventing glutamate excitotoxicity. Glutamate uptake is mainly accomplished by astrocytes which either convert glutamate to glutamine or feed it into their own metabolism. Normally, the astrocytic glutamate transporters are indirectly involved in the synaptic transmission termination by regulating the availability of glutamate for postsynaptic neuronal receptors. Moreover, they regulate the neurotransmitter release by controlling the amount of glutamate that reaches the presynaptic receptors. Finally, the astrocytic glutamate uptake protects neurons from hyperexcitability and subsequent excitotoxic damage by maintaining extracellular glutamate concentration under neurotoxic levels.

Although there are several membrane proteins capable of transporting glutamate, only members of EAAT are usually named as “glutamate transporters.” This name suggests that EAATs transport not only glutamate but also other excitatory amino acids. Five members of the family have been cloned and classified in the mammalian brain tissue—EAAT1-5. According to the solute carrier (SLC) terminology, EAAT1 corresponds to Slc1a3, EAAT2 corresponds to Slc1a2, EAAT3 to Slc1a1, EAAT4 to Slc1a6, and EAAT5 to Slc1a7 (Kanai and Hediger, 2004). In rodents, the EAAT1 analog has been termed as GLAST, EAAT2 as GLT-1 and EAAT3 as EAAC1.

The EAATs have been categorized as glial and neuronal, where the glial subfamily includes EAAT1 and EAAT2 and the neuronal subfamily consists of EAAT3-5. Nevertheless, the neuronal EAAT2 expression at the mRNA level was demonstrated quite a long time ago (Torp et al., 1997). The question of whether the neuronal expression has any functional relevance was partially answered by Petr and co-authors (Petr et al., 2015) who studied the function of EAAT2 expressed in excitatory synapses, employing conditional knockouts. Moreover, the contribution of presynaptic EAAT2 to the glutamate uptake has been acknowledged in hippocampal neurons (Furness et al., 2008).

The expression pattern of EAATs significantly differs in various brain regions. EAAT1 is preferably expressed in the cerebellum (Lehre and Danbolt, 1998) and a number of smaller regions, such as the inner ear (Takumi et al., 1997; Furness et al., 2008) the retina (Rauen et al., 1996; Lehre et al., 1997), and circumventricular organs (Berger and Hediger, 2000). In other CNS regions, EAAT2 prevails and is thus the major transporter responsible for glutamate uptake (Lehre and Danbolt, 1998). EAAT1 is often co-expressed with EAAT2, but they do not form hetero-oligomeric complexes. A higher density of EAAT1 and EAAT2 has been detected in membranes facing nerve terminals, axons and spines rather than in membranes facing capillaries, pia and stem dendrites (Chaudhry et al., 1995). Moreover, the expression pattern of EAATs changes during development. While EAAT1 predominates during the early development in radial glia and immature astrocytes, EAAT2 predominates in adulthood (Shibata et al., 1997; Kugler and Schleyer, 2004).

Similarly, neuronal transporters EAAT3 dominate in the hippocampus, while EAAT4s are abundantly expressed in cerebellar Purkinje cells (Furuta et al., 1997; Shashidharan et al., 1997; Dehnes et al., 1998; Massie et al., 2008). Both EAAT3 and EAAT4 are typically localized on the neuronal somas and dendrites and are not present on axonal terminals. EAAT5 is thought to be a retinal glutamate transporter and has not been identified in the brain so far (Eliasof et al., 1998).

The uptake of glutamate *via* EAATs is driven by the cotransport of 3Na^+^ and 1H^+^, followed by the counter-transport of K^+^. This stoichiometry provides enough energy for active glutamate transport into the cell against the steep concentration gradient. Positive charge entering the cell is compensated by an uncoupled flux of chloride ions (Cl^−^), thus the transporters also function as chloride channels (Fairman et al., 1995; Wadiche et al., 1995). Through this mechanism, L-glutamate, L- and D-aspartate, but not D-glutamate, can be transported (Drejer et al., 1982; Bender et al., 1998). Additionally, transmembrane domains of EAATs may form a water-permeable pore and thus they also contribute to water transport (MacAulay et al., 2001).

Since, EAATs play an essential role in regulating glutamatergic synapses, there are many neurological disorders that are related to the impaired activity of glutamate transport. During ischemia, insufficient energy supply results in decreased activity of Na^+^/K^+^ ATPase, which in turn causes a disruption of Na^+^ and K^+^ transmembrane gradients. Since glutamate transport *via* EAATs is coupled to the cotransport of Na^+^ and K^+^, the impairment of ion gradient leads to a reduced capacity of the EAATs and elevated extracellular glutamate concentrations. Finally, the ion gradient disruption may cause a reversal of transporter functioning, which contributes to an additional increase of glutamate levels in the ECS (Allen et al., 2004). The impairment of glutamate transport after ischemic injury contributes to the accumulation of extracellular glutamate and the neuronal damage associated with excitotoxicity. Therefore, the enhanced expression levels of astroglial glutamate transporters following brain ischemia may underlie the protective reaction triggered by high glutamate levels. Nevertheless, the results of studies analyzing changes in EAAT expression following an ischemic brain injury are inconsistent. The upregulation of EAAT1 and EAAT2 in the early phases of ischemia and its correlation with neuroprotection have been approved by many (Ganel et al., 2006; Weller et al., 2008; Arranz et al., 2010). On the contrary, other groups observed the downregulation of EAATs in different models of ischemic injury (Fukamachi et al., 2001; Rao et al., 2001; Chen et al., 2005; Moretto et al., 2005).

**Figure 3:**
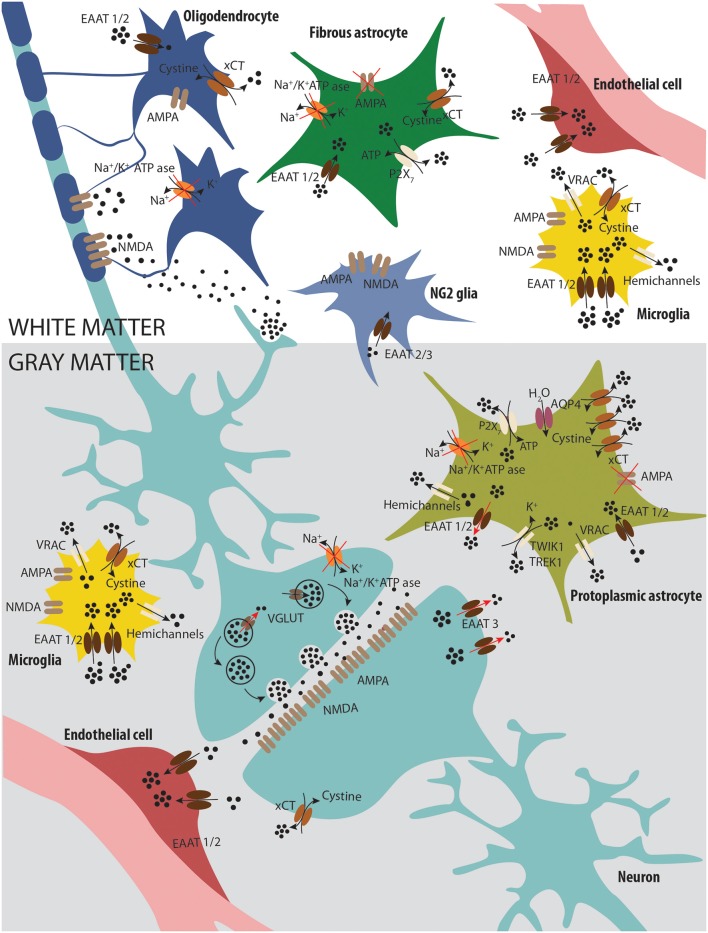
Various cell types in white and gray matter are hives of activity during ischemia-induced glutamate excitotoxicity. The scheme shows the main mechanisms that maintain or disrupt the homeostasis of glutamate (black dots) under ischemic conditions in white and gray matter. The location of individual receptors and transporters, as well as the direction of glutamate fluxes (arrows) in ischemia, are also depicted. In gray matter, glutamate is mainly released either from vesicles or *via* the reversal of glutamate transporters of neurons and astrocytes. The efflux through transporters is a result of Na^+^/K^+^ ATPase inhibition and subsequent collapse of the ion gradients. Since the operation of vesicular glutamate transporters (VGLUTs) depends on H^+^ ATPase activity, vesicles lose their glutamate upon adenosine triphosphate (ATP) depletion. The compromised astrocytic uptake of glutamate may be partially compensated by the activity of microglial and endothelial EAATs. Astrocytes and microglia also contribute to the excess of extracellular glutamate by overexpressing cystine/glutamate antiporters. Other contributors to the glutamate release were identified on the astrocytes, and comprise hemichannels, and TREK and TWIK channels. Swelling of astrocytes due to the activity of AQP4 channels, resulting in brain edema, opens VRACs that release glutamate to the extracellular space (ECS). The means of glutamate release in white matter differ from those identified in gray matter. Excitotoxicity is not enhanced by the reversal glutamate uptake, but *via* vesicular release from axons, which results in cytotoxic overactivation of myelinic N-methyl-D-aspartate (NMDA) receptors. Higher vulnerability/susceptibility of white matter to ischemia is a consequence of higher expression of AMPA and NMDA receptors on the cells of the oligodendrocytic lineage. Abbreviations: AMPA, α-amino-3-hydroxy-5-methyl-4-isoxazolepropionic acid; AQP4, aquaporin 4; EAAT1-3, excitatory amino acid transporters 1–3; NMDA, N-methyl-D-aspartic acid or N-methyl-D-aspartate; P2X_7_, ionotropic purinergic receptor 7; TREK1, two-pore-domain background K^+^ channel; TWIK 1, two-pore-domain K^+^ channel; VGLUT, vesicular glutamate transporter; VRAC, volume-regulated anion channels; xCT, cystine/glutamate antiporter.

Although it is generally accepted that astrocytes are the key players in glutamate uptake, which at synapses accounts for about 90% of total clearance, the contribution of other cell types cannot be overlooked. EAAT1 and EAAT2 are functionally expressed in microglia where they are involved in synaptic glutamate clearance (Noda et al., 1999; Morioka et al., 2008). Their expression enhances in the activated form of microglia following neuronal injuries (López-Redondo et al., 2000) and also in brain ischemia (Beschorner et al., 2007). Some studies show that under selected pathological conditions, the expression of astrocytic EAATs is decreased and conversely it increases in activated microglia (Xin et al., 2009). These findings suggest that under such conditions, or in restricted areas, microglia can adopt the role of astrocytes in glutamate uptake. In addition, oligodendrocytes express functional glutamate transporters with properties similar to those observed in astrocytes (Matute et al., 2006). Endothelial cells are another cell type, which significantly contributes to the regulation of extracellular glutamate concentration. These cells form one of the BBB layers and thus participate in the communication between brain tissue and the vascular system. Three members of the EAAT family, excitatory amino acid transporters 1–3 (EAAT1-3), have been detected in the abluminal membrane of the BBB (O’Kane et al., 1999). In brain pathologies such as ischemia, the increased concentration of extracellular glutamate activates the glutamate uptake *via* endothelial EAATs. Subsequently, the glutamate may be extruded across the luminal membrane into the bloodstream through another type of glutamate carrier, referred to as facilitative glutamate transporters (Hawkins and Viña, 2016). Finally, at least one study, performed in the retina, described an EAAT-mediated glutamate uptake by another brain cell type—pericytes (Akanuma et al., 2015). A scheme summarizing the mechanism of glutamate uptake in the ischemic CNS is shown in Figure 3.

## Ischemic Preconditioning

Alterations in the expression of glutamate transporters also appear to play a key role in the so-called ischemic preconditioning. This term refers to the cell/tissue adaptation to ischemic conditions after being exposed to “subtoxic” doses of stressors, such as hypo-/hyperoxic preconditioning, hypo-/hyperthermia, OGD or pharmacological preconditioning. Preconditioning results in a transient protective phenotype called ischemic tolerance, in which the tissue is more resistant to a subsequent more severe ischemic event. In the neural tissue, this phenomenon was first described in 1990 by Kitagawa and co-authors in several different areas of the gerbil brain (Kitagawa et al., 1990, 1991). Since then, many investigators have been engaged in finding molecular mechanisms that lie beneath the minimization of neuronal damage and the facilitation of regenerative processes. These mechanisms include complex biological cascades that are specific to the applied stimulus and employ both neuronal as well as glial pathways (for review, see Thushara Vijayakumar et al., 2016). These are mainly the immune response that involves the microglial activation of the pro-inflammatory cytokines, such as interleukins 1β and 6 (IL-1β, IL-6), and TNFα (Karikó et al., 2004), the nitric oxide synthase (NOS) cascade (Gidday et al., 1999) or the activation of certain enzymes, such as stress-activated kinases, cyclooxygenase-2 (COX-2; Iadecola et al., 2001; Colangelo et al., 2004) or sphingosine kinase 2 (SPK-2; Yung et al., 2012). Recent studies have shown that preconditioning can also activate changes at the gene level, which involves activation of molecules, such as transcription factors, transducers, sensors, and effectors, as well as numerous post-translational modifications. Genomic and proteomic analysis of the adult rat brain, following preconditioning induced by a 10-min transient middle cerebral artery occlusion (MCAO), showed for example upregulation of heat shock proteins or transforming growth factor-α (TGF-α; Dhodda et al., 2004).

In addition to these mechanisms, processes activated by increased glutamate concentration also play an important role (for review, see Krzyżanowska et al., 2014). This role includes the activation of NMDA receptors on the one hand and the regulation of EAATs on the other. NMDA activation was reported to mediate neuroprotection by inhibition of stress-activated c-Jun N terminal kinase (JNK), activation of extracellular signal-regulated kinase (ERK1/2) and protein kinase B (Akt1), and regulation of normal cAMP-responsive element-binding (CREB) activity (Miao et al., 2005; Soriano et al., 2006). In neuronal cortical cultures, tolerance was achieved with glutamate preconditioning and blocked by NMDA and AMPA receptor antagonists (Lin et al., 2008). Other studies have documented the decreased expression of glutamate receptors, in particular NMDA and AMPA, which reduced the excitotoxicity after ischemic preconditioning (Aizenman et al., 2000; Tanaka et al., 2002; Dave et al., 2005). Similarly, changes in the expression of glial GLT-1 have been also proposed as key mechanisms of ischemic preconditioning (Romera et al., 2007; Zhang et al., 2007; Gong et al., 2012). Moreover, OGD preconditioning performed in neuron/astrocyte co-culture revealed that the reversed operation of astrocytic GLT-1 was crucial to the development of neuronal ischemic tolerance (Kawahara et al., 2005). Recent studies show that GLT-1 expression and function of glutamine synthetase during preconditioning can be maintained *via* mechanisms including upregulation of Cx43 and inhibition of cellular kinase Src (Li et al., 2018). Another study suggests that p38 mitogen-activated protein kinase (MAPK) participates in the mediation of astrocytic GLT-1 upregulation during the induction of brain ischemic tolerance after ischemic preconditioning (Zhang et al., 2017).

## Mechanisms of Glutamate Release—The Contribution of Neurons and Glial Cells

In the CNS, glutamate is primarily released by excitatory neurons. The major pathway for its release from neurons comprises Ca^2+^-dependent exocytosis, which underlies fast synaptic transmission and, under physiological conditions, excitatory neurons represent a prime source of glutamate rises in the ECS. As already described above, the clearance of synaptically-released glutamate is one of the most important functions of astrocytes, which impedes glutamate excitotoxicity. Nevertheless, recent studies also point toward astrocytic function in releasing glutamate to the nearby neurons. Therefore, both the uptake and release of glutamate represent a precise mechanism for the fine-tuning of neuronal excitability, synaptic transmission, and plasticity under physiological conditions (Hamilton and Attwell, 2010; Araque et al., 2014; Gundersen et al., 2015; Allen and Eroglu, 2017). In CNS disorders, such as ischemia, trauma or neurodegeneration, the effective maintenance of glutamate homeostasis usually fails, while the glutamate release from different cellular types wins through, and results in detrimental glutamate excitotoxicity. Considering ischemic injury in the CNS, one should be conscious of the maturity of nervous tissue (perinatal, adult or aged), severity and type of ischemia (global vs. focal), region specificity (gray or white matter; cortex, hippocampus or corpus callosum) and phase of ischemia (acute or late), in which participation of cellular elements in glutamate release may differ. Two main phases were identified in severe cerebral ischemia *in vivo* (Astrup et al., 1977; Hansen and Nedergaard, 1988; Rossi et al., 2007). The first phase, taking a few minutes, is characterized by a gradual elevation of extracellular K^+^ concentration ([K^+^]_o_), with small changes in the concentration of other principal ions. The second phase is characterized by anoxic depolarization, loss of principal ionic gradients, and glutamate accumulation in the ECS. Furthermore, oxidative stress during post-ischemic reperfusion was proposed as an additional trigger of glutamate release (Tretter and Adam-Vizi, 2002).

### Neuronal Part in Glutamate Excitotoxicity

The main route by which mature axons release glutamate during acute ischemia primarily comprises vesicular release (Figure 3), which has been shown in white matter (Doyle et al., 2018), in cerebral ischemia *in vivo* (Katayama et al., 1991; Jabaudon et al., 2000), in a slice model of hippocampal ischemia (Andrade and Rossi, 2010), and in an *in vitro* model of ischemia (Fujimoto et al., 2004; Lee and Kim, 2015). The second route of glutamate release from neurons was described in severe brain ischemic injury by Rossi et al. (2000) who demonstrated that a reversed operation of neuronal glutamate transporters (Figure 3) plays a key role in generating the anoxic depolarization, that eliminates information processing in the CNS a few minutes after the onset of ischemia. However, several events that take place prior to glutamate release were described in the early phase of ischemia. In a slice model of hippocampal ischemia, Andrade and Rossi (2010) identified several early cellular cascades triggered by ischemia, such as Ca^2+^ entry and release from intracellular stores, actin filament depolymerization, and vesicular release of glutamate, which is dependent on actin dynamics but independent of [Ca^2+^]_i_ rise. Lee and Kim (2015) showed that the Ca^2+^ uptake *via* plasma membrane Na^+^/Ca^2+^ exchanger (NCX) underlies the ischemia-induced Ca^2+^ rises and the consequent increase in vesicular glutamate release from presynaptic terminals in the early phase of brain ischemia. All of these processes precede the anoxic depolarization by a few minutes.

In the acute phase of ischemia, which is characterized by anoxic depolarization and a massive increase in [Ca^2+^]_i_ mediated by both Ca^2+^ influx and Ca^2+^-induced Ca^2+^ release from intracellular stores, the vesicular release from presynaptic terminals further adds to the glutamate elevation. The importance of vesicular glutamate release from axons and its involvement in ischemia-induced myelin damage was recently documented by Doyle et al. (2018). Their findings contrast with previous reports describing reversal of glutamate uptake as a main contributor to glutamate excitotoxicity under ischemic conditions in the CA1 region of the neonatal hippocampus (Rossi et al., 2000), adult spinal cord (Li et al., 1999) or mouse optic nerve (Baltan et al., 2008). Their direct measurement of extracellular glutamate in white matter gave no evidence for significant ischemia-induced release *via* reversal of glutamate uptake. Ischemia-induced glutamate release pathways in the white matter of the brain thus clearly differ from those functioning in gray matter regions, such as the hippocampal CA1 or cortex. Doyle et al. (2018) showed that acute ischemic myelin injury is initiated by vesicular release from axons, which results in cytotoxic overactivation of myelinic NMDA receptors comprising GluN2C/D subunits, which are primarily incorporated into extra-synaptic NMDA receptors (Brickley et al., 2003).

We should also mention the cysteine-glutamate exchanger (xCT), which promotes glutathione synthesis by taking up cysteine and releasing glutamate, and which could possibly add to glutamate excitotoxicity evoked by ischemia and, consequently, to the overactivation of extrasynaptic NMDA receptors and neuronal death. In response to OGD, Soria et al. (2014) detected currents in pyramidal cortical neurons of rodent brain slices. These currents were sensitive to the pharmacological inhibition of the xCT but insensitive to the blockade of EAATs or vesicular glutamate release. Furthermore, xCT inhibition significantly attenuated neuronal damage (Soria et al., 2014). Since, xCTs were reported in neurons, but they were also found in astrocytes, oligodendrocytes, and microglia, it needs to be clarified whether xCT-released glutamate is of neuronal or glial origin (Figure 3).

### Astrocytes add Significantly to Glutamate Excitotoxicity

The mechanisms of glutamate release from astrocytes are still a matter of debate, especially in the field studying its role as gliotransmitter, and in conjunction with glutamate excitotoxicity in CNS disorders such as ischemia. Several studies suggest that astrocytes release glutamate *via* Ca^2+^-dependent vesicular release, by a mechanism analogous to that described in neurons, as reviewed recently by Savtchouk and Volterra (2018). Nevertheless, it is disputed by others, who emphasize that an increase in the astrocyte Ca^2+^ levels and the resulting glutamate release are non-specific and that astrocytes lack any vesicular release machinery (Barres, 2008; Fiacco and McCarthy, 2018). Furthermore, several non-vesicular release mechanisms have also been described (Nedergaard et al., 2002; Hamilton and Attwell, 2010; Gundersen et al., 2015).

The release of glutamate from vesicles can either be dependent on external Ca^2+^ or on Ca^2+^ release from intracellular stores (Bezzi et al., 2001, 2004). The presence of intracellular vesicles containing glutamate, VGLUTs 1 and 2 as well as specific vesicular proteins (v-SNAREs), were described in astrocytes by several research teams (Bezzi et al., 2004; Montana et al., 2004; Ni and Parpura, 2009; Bergersen et al., 2012). Recently, using single and double v-SNARE knockout mice, Schwarz et al. (2017) have proven that astrocytes express synaptobrevin II and cellubrevin on distinct vesicle populations and mediate glutamate and neuropeptide Y release through distinct, previously unrecognized, secretion pathways which may be essential in neuron-astrocyte communication. Different experimental strategies that demonstrate a functional role of vesicular release from astrocytes were summarized by Bohmbach et al. (2018).

In cerebral ischemia, the non-vesicular glutamate release in astrocytes is primarily represented by a reversed operation of glutamate transporters, which occurs under the conditions upon intracellular ATP depletion. Since the energy requirements for glutamate uptake are extremely high, the reversal of glutamate transport is preceded by a decline in astrocytic glutamate uptake, which signifies the earliest astrocytic contribution to the development of glutamate excitotoxicity, together with the already reversed glutamate uptake in neurons (Rossi et al., 2000). It was shown that the expression of both glutamate transporters (EAAT1 and EAAT2) in astrocytes is rapidly reduced following hypoxia and ischemia, which leads to further extrasynaptic glutamate accumulation (Sheldon and Robinson, 2007; Ketheeswaranathan et al., 2011). Furthermore, the ATP-dependent conversion of glutamate into glutamine by astrocytic glutamine synthetase turns out to have been impaired during energy failure, thus elevating the intracellular glutamate concentrations in astrocytes, which subsequently leads to reduced glutamate uptake or even enhanced glutamate release by reversed uptake (Lipton and Rosenberg, 1994). As reviewed in detail by Pajarillo et al. (2019), the expression and function of glutamate transporters during ischemia may be dysregulated at different levels, ranging from either positive or negative transcriptional regulation, RNA splicing, epigenetic modulation, such as DNA methylation or histone modification, to post-translational glycosylation and phosphorylation.

Apart from glutamate transporters, several ion channels that may release glutamate from astrocytes were already proposed. Interestingly, under ischemic stress, Liu et al. (2006) demonstrated the participation of maxi-anion channels and volume-sensitive outwardly rectifying anion channels (VRACs) in glutamate release from mouse astrocytes, with the predominant contribution of maxi-anion channels. They also found that the contribution of gap junction hemichannels, vesicle-mediated exocytosis, and reversed operation of the Na^+^-dependent glutamate transporters, was none or minor (Liu et al., 2006). Employing transient MCAO, together with the microdialysis and pharmacological inhibition of GLT-1 and VRACs, Feustel et al. (2004) demonstrated that VRACs are the predominant contributors to glutamate release in the ischemic cortical penumbra; this contrasts with severely affected ischemic regions, in which the reversal of glutamate transport plays a major role. Such evidence supporting VRACs as astrocytic glutamate-releasing channels were indirect and based on pharmacological inhibitors, which may affect the function of other membrane proteins, including those directly involved in the glutamate transport (Bowens et al., 2013). Nevertheless, the latest work of Yang et al. (2019) revealed astrocytic VRACs as major candidates mediating glutamate release in FCI. They took advantage of recent findings, which revealed that the leucine-rich repeat-containing protein 8A (LRRC8A, also named SWELL1) and its four other associated homologs (LRRC8B-E) form heteromeric VRACs (Voss et al., 2014; Schober et al., 2017; Osei-Owusu et al., 2018). In a well-designed approach, which employed a conditional knockout of *Swell1* in astrocytes in an experimental model of stroke, Yang et al. (2019) proved that the *Swell1^−/−^* mice had significantly smaller infarct volumes and better scores for overall neurological function than the control mice. In contrast to the role of VRACs in astrocytic glutamate release, their activation in neurons was shown to mediate Cl^−^ influx and further contribute to cell damage by promoting neuronal swelling (Inoue and Okada, 2007; Zhang et al., 2011).

Upon G protein-coupled receptor activation, Ca^2+^-dependent anion channel Bestrophin 1 and two-pore domain K^+^ channels TWIK1 and TREK1 were also suggested to participate in glutamate release (Woo et al., 2012; Ryoo and Park, 2016). Nevertheless, a markedly increased TREK1 expression in the hippocampal CA1 region and cerebral cortex after FCI was rather attributed to their neuroprotective function (Pivonkova et al., 2010; Wang et al., 2012; Pivonkova and Anderova, 2017). Clarification of the role of these channels in glutamate release will require the generation of conditional knockout mice, in which TREK1 or TWIK1 channels are removed specifically in astrocytes, thus providing a suitable system to assess their participation in neuroprotection or glutamate excitotoxicity (Heurteaux et al., 2004; Wu et al., 2013).

In response to extracellular ATP, the P2X_7_ purinergic receptors may shift from rapid-gating channels selective for small cations to more slowly developing “dilated pore” conformations permeable to molecules up to 900 Da (North, 2002) and thus facilitate glutamate release under pathological conditions (Duan et al., 2003). In the CNS, these receptors were described in astrocytes, microglia, oligodendrocytes, and neurons (Hamilton et al., 2008; Grygorowicz et al., 2010; Burnstock, 2017); however, their precise role in glutamate excitotoxicity is not yet fully elucidated.

Astrocytes and neurons also express several large pore hemichannels that may open in response to various stimuli, allowing cytoplasmic molecules such as ATP and glutamate permeate (Hansen et al., 2014). Several studies revealed that the expression of astrocytic Cx43 is increased after hypoxia/ischemia and that Cx43 may play an important role in cell death and neuronal damage (Davidson et al., 2013; Ma et al., 2018). The astrocytic hemichannels are activated *via* increased Ca^2+^ levels and inflammatory factors, such as IL-1β, epidermal growth factor (Morita et al., 2007), or TNFα (Giaume et al., 2013). Their activation then results in the uncontrolled release of ATP and glutamate. Key membrane proteins participating in astrocytic glutamate release are summarized in Figure 3. Nevertheless, their exact role in astrocytic and neuronal (patho-) physiology is still not well defined.

There is no doubt about the importance of astrocytes in maintaining CNS health, while their role in CNS disorders seems to be ambiguous. The failure of their crucial functions has emerged to be one of the important contributing factors of the several CNS disorders situating them to the position of “foes.” On the other hand, they are able to protect or support neighboring cells, especially during the early phases of ischemia, which marks them as “good guys.” Thus, defining astrocyte heterogeneity in the healthy, but also ischemic, CNS may enable us to better understand their roles and to support “beneficial” astrocytes and suppress those damaging the CNS.

### Oligodendrocytes

Oligodendrocytes and axons are the main targets of ischemic white matter injury. Myelinating oligodendrocytes and their precursor cells are particularly vulnerable to energy deprivation (Fern and Moller, 2000). In the conditions of OGD, the earliest changes are visible on oligodendrocytes, especially the swelling of their soma, which extends to myelin processes and subsequently results in axonal beading (Baltan, 2014). Oligodendrocytes express functional glutamate transporters with properties similar to those observed in astrocytes and may also operate in the reverse mode, releasing glutamate into the ECS under conditions of energy deprivation (Matute et al., 2006). The main transporter expressed by oligodendrocytes is GLAST (EAAT1; Figure 3), while a subpopulation of adult oligodendrocyte progenitors expresses the EAAC1 (EAAT3), which is also present in neurons (Domercq et al., 1999).

### Microglia and Glutamate Release

Upon activation, microglia may release high amounts of the neurotransmitter glutamate, thus also contributing to excitotoxic injury during brain hypoxia-ischemia. It has been shown that microglia possess two main systems for glutamate transport, GLT-1 for transport into the cells and the xCT system for transport out of the cells. Glutamate taken up through GLT-1 is used for direct incorporation into glutathione and to supply the intracellular glutamate pool to allow cystine uptake through xCT (Bridges et al., 2012; Persson and Rönnbäck, 2012). The presence of VRACs and their contribution to glutamate release in microglia (Figure 3) was demonstrated by exposing rat microglia to hypoosmotic stress, H_2_O_2_, or ROS-triggering zymosan; this potently increased the swelling-activated glutamate release in these cells and was sensitive to VRAC blocker, DCPIB (Harrigan et al., 2008). Similarly to astrocytes, activated microglia release excessive glutamate through gap junction hemichannels (Takeuchi et al., 2006) and their importance in glutamate excitotoxicity were described by Umebayashi and co-authors, as the blockade of these channels by INI-0602 was protective in secondary spinal cord injury (Umebayashi et al., 2014). Recently, a signaling pathway controlling glutamate release from human microglia during hypoxia was revealed by Socodato et al. (2018). They showed that a hypoxia-mediated redox imbalance promotes the release of Ca^2+^ from the endoplasmic reticulum and activates the non-receptor protein tyrosine kinase Src at the plasma membrane; this enhances the glutamate permeability of microglial gap junctions. Microglia can influence glutamate homeostasis also indirectly by releasing factors such as ROS, TNFα, and IL-1β, which impairs the function of glutamate transporters in other glial cells (Domercq et al., 2007).

### ATP Release

Impact similar to the elevation of glutamate in the ECS was observed in the uncontrolled increase of extracellular ATP. Anoxic depolarization, which occurs during ischemia, triggers ATP release from neurons and glial cells leading to a marked elevation of extracellular ATP concentration (Dale and Frenguelli, 2009), which leads to Ca^2+^ overload, tissue excitotoxicity, and amplification of the inflammation (Domercq et al., 2010; Kim Y. et al., 2018). During ischemia, ATP release originates from multiple sources, such as vesicular release from neurons (Orlando et al., 2014), exocytosis from microglia (Imura et al., 2013) and astrocytes (Hamilton and Attwell, 2010), hemichannels that can release ATP from glial cells (Montero and Orellana, 2015), or by opening of P2X_7_ ionotropic receptors that form a large pore allowing the ATP release from astrocytes (North, 2002; Yan et al., 2010). Additionally, cell death and membrane damage cause an additional massive release of glutamate and ATP, aggravating the initial ischemia-induced damage (Matute and Ransom, 2012). ATP acts on purinergic receptors and exerts a cytotoxic role in the CNS. Among P2X receptors, most evidence indicates that P2X_7_ receptors contribute to ischemia-induced damage *via* marked Ca^2+^ entry into the neurons and glia and facilitation of glutamate release (Arbeloa et al., 2012; Zeng et al., 2012; Eyo et al., 2013). In oligodendrocytes, the sustained P2X_7_ receptor activation induces cell death, myelin damage, and white matter injury (Matute et al., 2007; Matute, 2008; Domercq et al., 2010). For a detailed review covering purinergic signaling in brain ischemia, see Pedata et al. (2016).

### Glutamate-Induced Brain Edema

Increases in the extracellular glutamate concentration have been put into context with cerebral edema, namely the so-called cytotoxic edema of astrocytes. Brain edema may be defined as an increase in the brain tissue volume resulting from the abnormal accumulation of water. Usually, three types of edema are distinguished according to the mechanisms responsible for the water accumulation in tissue—cytotoxic, ionic and vasogenic edema. In the early phases of ischemia, cytotoxic edema occurs as a result of the accumulation of osmotically active ions within the cell, followed by the entry of water from the ECS. This is followed by the formation of ionic edema as a result of an extracellular ionic and water level decrease, creating new gradients between plasma and interstitial fluid and driving trans-capillary fluxes of ions and osmotically obligated water. Vasogenic edema is a consequence of the BBB disruption, which leads to the water/macromolecules flux from the bloodstream to the brain ECS and it usually accompanies progressed stages of brain ischemia or other pathologies. This issue is discussed in detail in the excellent reviews by Pasantes-Morales and Vázquez-Juarez (2012) and Thrane et al. (2014). Here, we only focus on the mechanisms of cytotoxic stadium of the brain edema, which is related to the increased glutamate levels and the impaired glutamate uptake.

*Cytotoxic (cellular) edema* accompanies pathological states that are related to the ionic disbalance, such as hyponatremia, ischemia, brain trauma or hepatic encephalopathy. Astrocytes are key contributors to the generation of cellular edema due to their relationship with the cerebral vasculature and their prominent homeostatic functions. Nevertheless, the ability of cytotoxic swelling has also been described in other cell types, namely neurons and endothelial cells (Panickar et al., 2015; Jayakumar et al., 2018) For review, see the work of Jha et al. (2019). The swelling of astrocytes is the result of a number of mechanisms that, however, depend on specific pathological conditions and may also vary locally. In general, astrocytic swelling is triggered by the uptake of osmotically active substances, so-called primary drivers, which are normally more concentrated outside the cell and extruded from the cell by active transport. Their movement results in the creation of chemical gradients for other molecules further termed secondary participants, for which no pre-existing electrochemical gradients normally exist, and which move in order to maintain electrical and osmotic neutrality (Simard et al., 2006). For example, increases in intracellular Na^+^ concentration ([Na^+^]_i_) lead to the entry of Cl^−^ and water, where Na^+^ is a primary driver and Cl^−^ and water are secondary participants. In ischemia, the key mechanisms include the water channel, aquaporin-4 (AQP4; Manley et al., 2000), the Na^+^-K^+^-Cl^−^ cotransporter 1 (NKCC1; Su et al., 2002a,b), the sulfonylurea receptor 1 (SUR1)-regulated non-selective cation channels (NCCa-ATP; Simard et al., 2006; Stokum et al., 2018), the Na^+^-dependent EAATs (Schneider et al., 1992), and finally, Donnan cell swelling including Cl^−^ and K^+^ channels (Kimelberg, 2005). The specific mechanisms depend on the distance from the ischemic core. In the ischemic core, rapid depletion of energy substrates leads to the inhibition of Na^+^/K^+^ ATPase and subsequent collapse of the ion gradient—a marked increase in the [Na^+^]_i_ and [Ca^2+^]_i_ and extracellular K^+^ concentrations. The resulting membrane depolarization then creates an inwardly directed driving force for Cl^−^ *via* anion channels, which in turn causes the additional influx of Na^+^ and K^+^ in order to achieve charge neutralization. This mechanism of cell swelling has been coined in the literature as Donnan cell swelling (Kimelberg, 2005; Wilson and Mongin, 2018). Moreover, the ATP sensitive SUR1-transient receptor potential melastatin 4 channels, which can operate without energy supply, play a significant role in the astrocytic swelling located in the ATP-depleted areas (Chen and Simard, 2001; Chen et al., 2003). In the penumbra, where the energy supply persists and a gradual increase in K^+^ and glutamate concentrations occurs, the NKCC1 and Na^+^-dependent glutamate transporters are the main mechanisms involved in astrocytic swelling.

Considering the topic of this review, we only focus therein on mechanisms that are activated by increased glutamate levels or, conversely, contribute to the release of glutamate into the ECS (Figure 3). Glutamate may affect the astrocytic swelling indirectly through the stimulation of neuronal activity, or directly *via* astrocytic glutamate receptors and transporters. In the latter case, astrocyte swelling is either caused by the accumulation of glutamate alone or by ions, whose transport is activated by the binding of glutamate to mGluRs.

### Glutamate Transporters

The activation of Na^+^-dependent glutamate transporters results in the influx of glutamate molecules together with 3 Na^+^. These both act as primary drivers and are followed by water movement into the cell (Chan and Chu, 1990; Schneider et al., 1992). As we already mentioned, the glutamate uptake *via* EAATs is a process dependent on the electrochemical gradient of Na^+^ and hence on the activity of Na^+^/K^+^ ATPase. Therefore, this mechanism occurs in areas where energy substrates are not completely depleted. In addition, in the case of energy depletion, these transporters can function in the reversed mode and thus contribute to glutamate release as also mentioned above.

### Metabotropic Glutamate Receptors

The idea of astrocytic swelling induced by the activation of mGluRs was supported by studies that showed that swelling of cultured astrocytes was induced by mGluR agonists trans-ACPD and L-AP4 (Hansson, 1994; Yuan and Wang, 1996). Alternatively, other mGluR agonists, e.g., ibotenic acid, did not induce any swelling and, moreover, mGluR antagonists MCPG and MAP4 did not prevent the swelling (Bender et al., 1998). The activation of mGluRs leads to the [Ca^2+^]_i_ increase and finally, to the opening of K^+^ channels (Hansson, 1994; Bender et al., 1998). Moreover, it has been shown, that one of the astrocytic glutamate receptors, mGluR5, forms a functional tripartite complex together with AQP4 and Na^+^/K^+^ ATPase, which has an important function in the regulation of water and K^+^ homeostasis in the brain (Illarionova et al., 2010). As all components of this system are involved in the glutamate-induced swelling (Bender et al., 1998), it is possible that the whole system plays a role in astrocyte swelling (Stokum et al., 2015).

### Ionotropic Glutamate Receptors

Since NMDA receptors mediate the transfer of Na^+^ into cells, there is a possibility that they represent, in addition to glutamate transporters and metabotropic receptors, another mechanism responsible for the cytotoxic swelling activated by increased glutamate concentrations (Winkler et al., 2016). In astrocytic cultures, this possibility has been confirmed using specific NMDA receptor inhibitors such as ketamine or dizocilpine (MK801; Chan and Chu, 1990; Chan et al., 1990; Hansson, 1994).

### Glutamate-Induced K^+^ Uptake by K^+^ Channels

The uptake of K^+^ is most likely triggered by the activation of Na^+^/K^+^ ATPase as a result of glutamate cotransport together with Na^+^. As the glutamate-induced swelling is sensitive to the specific K^+^ channel blockers, namely Ba^2+^ and tetraethylammonium (TEA), it is likely that delayed outwardly and inwardly rectifying K^+^ channels are involved (Bender et al., 1998).

### Regulatory Volume Decrease (RVD)

As uncontrolled volume changes are harmful to cells and may eventually lead to cell death, mechanisms controlling the cell volume are essential for physiological cell functioning. The majority of cells possess regulatory volume mechanisms, which involve specific membrane transport and/or metabolic processes leading to compensatory changes in the intracellular osmolyte concentrations and thus returning cell volume to the normal resting state. The generated osmotic gradients subsequently drive water across the membrane by diffusion through the bilayer or through AQPs. The recovery of cell volume after swelling is accomplished by the extrusion of the major intracellular ions (K^+^ and Cl^–^) and organic osmolytes, such as taurine and amino acids (Sanchez-Olea et al., 1993a,b; Vitarella et al., 1994). The efflux of Cl^−^ and also the organic osmolytes during regulatory volume decrease (RVD) are mainly mediated by VRACs (Abdullaev et al., 2006). The conductivity of VRACs for small organic molecules is important in this context, mainly because these substances (e.g., glutamate, aspartate) are also major CNS neurotransmitters. Thus, the release of these molecules affects brain functions, and in the case of glutamate, it dramatically contributes to the glutamate toxicity as suggested by Kimelberg et al. (1990). For more details on this subject, see Kimelberg (2005) and Mongin (2016).

## Pathological Consequences of Glutamate Excitotoxicity

In general, an excess or prolonged exposure of neurons and glia to high levels of extracellular glutamate during ischemia both lead to a massive Ca^2+^ entry into the cells of both gray and white matter (Danysz and Parsons, 2003; Van Damme et al., 2007). Such excessive [Ca^2+^]_i_ initiates a series of molecular neurotoxic cascades, including the activation and overstimulation of proteases, lipases, phosphatases, and endonucleases (Orrenius et al., 2003). This leads to the activation of several signaling pathways, mainly causing an overproduction of free radicals, mitochondrial damage, cell membrane disruption, and DNA fragmentation, which all culminate in apoptotic or necrotic cell death (Figure 2; Rama and García, 2016).

### Overactivation of Glial Glutamate Receptors

Glutamatergic signaling through glutamate receptors forms a major component of excitatory synaptic transmission in the CNS. However, glutamate release in the CNS is not exclusive to synaptic terminals but also arises from unmyelinated axons and glial cells under both physiological and pathophysiological conditions (Ceprian and Fulton, 2019). Glial cells express both types of glutamate receptors—ionotropic (NMDA, AMPA, and kainate) and metabotropic in gray and white matter. For reviews, see D’Antoni et al. (2008), Dzamba et al. (2013), Ceprian and Fulton (2019) and Fern and Matute (2019).

***In astrocytes***, the most probable composition of NMDA receptors seems to be tri-heteromeric, containing GluN1, GluN2C/D and GluN3 subunits (Palygin et al., 2011); however, the subunit composition varies regionally as well as in different developmental stages of nervous tissue and in various pathological states. Functional studies (Shelton and McCarthy, 1999; Lalo et al., 2006; Serrano et al., 2008) suggest that astrocytic NMDA receptors are primarily involved in neuron-astrocyte communication. It has been shown that brain ischemia results in the increased expression of NMDA receptors in astrocytes (Gottlieb and Matute, 1997; Krebs et al., 2003). Functional AMPA receptors of astrocytes are present in the majority of the CNS regions except for the hippocampus. The subunit composition of astrocytic AMPA receptors differs from region to region, leading to the variability in their permeability to Ca^2+^ (Matthias et al., 2003). Astrocytic AMPA receptors do not appear to be vulnerable to pathological conditions associated with glutamate-mediated excitotoxicity because of their low Ca^2+^ permeability (Li and Stys, 2000). These findings, and similar observations of excitotoxic resistance in astrocytes (Rothman, 1984), are supported by RNA-seq data, showing the dominance of GluA2 (inhibits permeability of Ca^2+^) in both cortical and hippocampal astrocytes *in situ* (Mölders et al., 2018). This excitotoxic resistance of astrocytes is weakened in white matter fibrous astrocytes, but there is currently no evidence that this is related to the expression of glutamate receptors (Fern and Matute, 2019).

***Oligodendrocytes*** are known to be extremely sensitive to OGD and their vulnerability changes during development and aging. Similarly to neurons, the enormous Ca^2+^ entry induced by glutamate excitotoxicity occurs *via* activation of different glutamate receptors. This process is involved in the death of oligodendrocytes in ischemic damage of gray as well as white matter (Ransom and Baltan, 2009; Matute, 2011). The subunit composition of glutamate receptors and their localization in oligodendrocytes (soma, processes, myelin) predispose them to the susceptibility to ischemic injury as their AMPA receptors are especially permeable to Ca^2+^ (Káradóttir and Attwell, 2007) and their NMDA receptors are only weakly blocked by Mg^2+^, allowing them to produce considerable currents at resting membrane potential (Káradóttir et al., 2005). Moreover, oligodendrocytes also express receptors of all three groups of metabotropic glutamate receptors, but their levels are developmentally regulated and are very low in mature cells of this lineage (Deng et al., 2004). The overactivation of AMPA and kainate receptors leads to the demise of oligodendrocytes and primary and/or secondary myelin destruction as a consequence of massive influx of Ca^2+^ (McDonald et al., 1998; Alberdi et al., 2002; Sánchez-Gómez et al., 2011). Also, it has been shown that ischemia modeled *in vitro* leads to the NMDA receptor activation, resulting in Ca^2+^ increases in oligodendrocytes that are abolished by NMDA antagonists, such as memantine, D-AP5, or MK-801 (Salter and Fern, 2005; Micu et al., 2006, 2007). Salter and Fern (2005) have also reported that the activation of NMDA receptors results in the fast Ca^2+^-dependent morphological changes, which are manifested by detachment and disintegration of oligodendroglial processes and formation of swellings, and that blocking the NMDA receptors prevents injury to oligodendrocytic processes.

***Oligodendrocyte precursor cells (NG2 glia)*** and immature oligodendrocytes are very sensitive to transient OGD (Fern and Moller, 2000). Thus, simulated ischemia in young animals induces an inward current in oligodendrocytes that are partly mediated by NMDA and AMPA/kainate receptors (Káradóttir et al., 2005). The sensitivity to hypoxia-ischemia damage has been correlated to the maturation of oligodendrocytes in several species (Riddle et al., 2006; Segovia et al., 2008; Buser et al., 2010) including human (Back et al., 2002), with pre-oligodendrocytes being identified as particularly vulnerable. Importantly, within the oligodendrocyte lineage, pre-oligodendrocytes in developing white matter exhibit the greatest abundance of GluA4 (Talos et al., 2006), a Ca^2+^-permeable subunit, highly expressed in neural cells exhibiting vulnerability to excitotoxic death (Page and Everitt, 1995).

***Microglia*** express members of all ionotropic and metabotropic glutamate receptors. Activation of ionotropic receptors leads to enhanced release of TNFα, IL-1 and NO (Noda et al., 2000; Murugan et al., 2011). Specifically, increased expression of AMPA receptors in hypoxic white matter sensitizes microglia to glutamate, leading to a regulatory response that reduces protective IGF-1 release, while increasing the release of inflammatory mediators (Sivakumar et al., 2010). Regarding metabotropic glutamate receptors, different subunits of metabotropic groups I (mGluR5), II (mGluR2 and 3), and III (mGluR4, 6, and 8, but not mGluR7) are expressed by microglia and regulate microglial transformation into neuroprotective (*via* group III mGluRs) or neurotoxic (*via* group II mGluRs) phenotypes (Pocock and Kettenmann, 2007).

## Periventricular Leukomalacia

The specific case of overactivation of glial glutamate receptors occurs in premature white matter injury. The premature brain undergoes critical developmental events as neuronal migration, growth of axons and dendrites, synaptogenesis, development of the vascular system, and myelination. The abundance of glutamate receptors in the white matter is a key element for these processes, but also a reason for increased vulnerability to possible excitotoxic conditions (Pregnolato et al., 2019). The presence of glutamate receptors is developmentally regulated. In the case of pre-oligodendrocytes, AMPA receptors are expressed, while in mature oligodendrocytes, NMDA receptors prevail. The overstimulation of non-NMDA receptors results in the rapid cell death, while overstimulation of NMDA receptors leads to a loss of cellular processes (Deng and Poretz, 2003; Deng et al., 2004, 2006). Due to this fact, hypoxia in the premature brain leads to white matter injury termed periventricular leukomalacia (PVL), which manifests as cerebral palsy and cognitive/behavioral deficits (Wilson-Costello et al., 2007). The injury of white matter is characterized by focal periventricular necrosis, with axonal damage and macrophages along with diffuse inflammation, with reactive astrocytes and activated microglia in the surrounding white matter. Pre-oligodendrocytes, which are more vulnerable than immature neurons or mature oligodendrocytes, are most affected (Haynes et al., 2003). The unique feature of the PVL is an arrest in the development of oligodendrocytes at the pre-oligodendrocyte stage, leading to the abnormal myelination (Volpe et al., 2011). Damage to pre-oligodendrocytes and hypomyelination is unlikely to be solely due to autonomous cell-intrinsic vulnerability. Numerous studies have demonstrated that microglial activation and astrogliosis play important roles in triggering white matter injury in PVL (Liu et al., 2013). Activated microglia mediate the death of pre-oligodendrocytes by two distinct mechanisms in a time-dependent manner. The early phase of cell damage occurs within 24 h and is mediated by NO-dependent oxidative damage. The delayed cell death, mediated by cytokines, is evident at 48 h (Pang et al., 2010; Falahati et al., 2013). Similarly, astrocytes induce apoptosis of oligodendrocytes by producing inflammatory cytokines such as TNFα and IL-1β (Deng et al., 2014). Currently, there is no specific treatment for PVL, but many studies try to find specific agents to attenuate white matter damage by preventing cell death of pre-oligodendrocytes. Pre-treatment with MgSO_4_ attenuated the loss of oligodendrocyte markers, such as myelin basic protein (MBP) and oligodendrocyte transcription factor (Olig2), in ipsilateral white matter and decreased the number of microglia, preventing thus cell death of pre-oligodendrocytes (Seyama et al., 2018). Another studied approach to ameliorate PVL was to transplant human oligodendrocyte progenitors. The results indicate that the transplanted cells restored neurobehavioral functions by preventing axonal demyelination and that human oligodendrocyte progenitor cells could be a suitable candidate for cell therapy of PVL (Kim T. K. et al., 2018).

## Oxidative Stress

Free radicals are the result of normal cellular function and metabolic activity of the cell. They could be any chemical species (atoms, molecules) capable of independent existence, and having one or more unpaired electrons. The most common free radicals induced by excitotoxicity are molecules derived from oxygen and NO, called ROS (superoxide radicals and hydroxyl radicals) and reactive nitrogen species (RNS; NO, peroxynitrite), respectively. Free radicals are highly reactive and can directly oxidize and damage macromolecules, such as proteins, lipids, and DNA (Sugawara et al., 2002; Kalogeris et al., 2014). Oxidative stress represents a condition, in which the production of free radicals exceeds the capacity of the antioxidant defenses to keep the levels below the toxic threshold. The cell uses different antioxidant defenses, including superoxide dismutase, catalase, glutathione detoxification pathways, and thioredoxin detoxification pathways (Kudin et al., 2012).

In general, neurons and glial cells possess the ability to defend against oxidative stress, but astrocytes display the highest basal levels of glutathione in the CNS, which predetermines their ability to remove high amounts of ROS and RNS (Sun et al., 2006). Furthermore, the glutathione system is regulated by the inducible nuclear factor erythroid-2-related factor 2 (Nrf2; Bell et al., 2015). Astrocytes, compared to neurons, have enhanced Nrf2 that upregulates antioxidant genes (NQO1, glutathione reductase, TXNRD1, glucose-6-phosphate dehydrogenase, malic enzyme, transketolase, and transaldolase) to protect astrocytes and also neurons (Lee et al., 2003; Shih et al., 2003). They are also capable of modulating the microglial inflammatory response, providing negative regulation by releasing an astrocyte-based factor that induces the microglial Nrf2 activity and increases the expression of antioxidant heme oxygenase-1. These antioxidant products suppress ROS/RNS and return microglia to the resting state (Min et al., 2006). Astrocytes also protect the brain against ischemia-associated oxidative stress by producing ROS scavenger metallothionein-II (Trendelenburg et al., 2002; Waller et al., 2018).

Oligodendrocytes are less vulnerable to oxidative stress than neurons but much more than other glial cells (Husain and Juurlink, 1995). Such vulnerability could originate from the highest levels of immobilized, protein-bound iron, which can be a potent cytotoxin by catalyzing the conversion of hydrogen peroxide to hydroxyl radicals *via* the Fenton reaction (Connor and Menzies, 1996). Moreover, when compared to astrocytes, oligodendrocytes possess less than half of the glutathione content, which leads to less effective peroxide scavenging due to the lower activity of glutathione peroxidase (Juurlink et al., 1998). They also have a high metabolic activity that requires a high consumption of oxygen and ATP, which generates more ROS (Mifsud et al., 2014). Oxidative stress is the leading cause of hypomyelination and mature oligodendrocyte deficiency because it increases the expression of genes known to inhibit oligodendroglial differentiation (French et al., 2009). Oligodendrocyte precursor cells are even more susceptible to oxidative stress than oligodendrocytes. The higher vulnerability is due to an upregulation of AMPA receptors (Itoh et al., 2002) and transiently increased expression of the glutamate transporter EAAT2 (Desilva et al., 2007). A recent study showed that the antioxidant signaling of OPCs is regulated by miR-146b-5p, enhancing the activation of Nrf2 signaling, which attenuates apoptosis and ROS production (Li et al., 2019). Catalpol, a potent antioxidant and free radical scavenger acting through the ERK1/2 signaling pathway, has also been shown to positively affect NG2 glia (pre-myelinating oligodendrocytes) after oxidative stress (Cai et al., 2016).

## Mitochondrial Dysfunction

The ischemia-induced excitotoxicity results in mitochondrial malfunctioning due to the damage to mitochondrial proteins, membranes, and DNA, thus impairing the ability of mitochondria to synthesize ATP and carry out their wide range of metabolic functions (Rama and García, 2016). The permeability transition pore (mPTP) in the mitochondrial membrane develops in response to Ca^2+^ overload and enables the release of pro-apoptotic factors. Such factors include cytochrome C, apoptosis-inducing factor (AIF) and Smac/Diablo, which participate in the activation of intrinsic apoptotic signaling (Yang et al., 2018). Ischemia also triggers the depolarization of the mitochondrial membrane, resulting in excessive ROS production, decreased ATP generation and PTEN-induced putative kinase 1 accumulation (Lazarou et al., 2015). As a consequence of ATP reduction, damaged mitochondria are repaired through the fusion with healthy mitochondria, or mitochondrial fission enables the segregation of damaged mitochondria and subsequent elimination *via* mitophagy (Liu et al., 2018). Stressed or dying cells with dysfunctional mitochondria might acquire healthy mitochondria through tunneling nanotubes to another cell type (Torralba et al., 2016). A recent study also showed that neurons already under physiological conditions release mitochondria that are internalized and degraded by adjacent astrocytes (Davis et al., 2014). Moreover, after stroke, astrocytes can release functional mitochondria towards neurons by a Ca^2+^-dependent mechanism. This neuroglial crosstalk contributes to neuroprotective mechanisms after stroke, since the suppression of this mitochondrial transfer worsens the neurological outcome (Hayakawa et al., 2016). Interestingly, a subset of mitochondria (35%) faces extreme Ca^2+^ overload, which acts as a “point of no return” and causes delayed excitotoxic cell death despite the fact that the majority of undamaged mitochondria still maintain normal functions (Pivovarova et al., 2004).

## Calpain Excitotoxicity

Additionally, the Ca^2+^ overload triggers the hyperactivation of calpains, Ca^2+^-dependent proteases that under pathological conditions add to the ischemia-induced excitotoxic damage. Calpains cleave the NCX3, which further leads to extra Ca^2+^ accumulation in neurons (Pignataro et al., 2004; Bano et al., 2005). Subsequently, Ca^2+^ activates a protease that transforms xanthine dehydrogenase into xanthine oxidase, an important enzyme in the production of free radicals (Gagliardi, 2000).

## Cell Death

Cell death is induced by many mechanisms: necrosis, apoptosis, autophagy, oncosis, necroptosis, etc., depending on the onset and the severity of ischemia (Fricker et al., 2018). Necrosis occurs mainly in the ischemic core as a result of a severe deficit or absence of oxygen and glucose. On the contrary, apoptosis takes place in the ischemic penumbra, where certain levels of oxygen and glucose remain, still maintaining the functional activity of cells (Rama and García, 2016). The difference is also in the time window when these mechanisms occur. Necrosis is mainly detected during the first post-ischemic minutes, whereas apoptosis develops hours or days later. Apoptosis, unlike necrosis, requires the integrity of the plasma membrane and a sufficient amount of ATP (Radak et al., 2017). The dichotomy between necrosis in the ischemic core and apoptosis in the ischemic penumbra is not strict as apoptotic death has been also shown in the ischemic core and vice versa (Onténiente et al., 2003).

## Apoptosis

Apoptosis can be activated by two general pathways: the extrinsic pathway is initiated by the death receptors, such as TNF receptors, CD95/Fas or TRAIL expressed on the cell surface. These ligand-activated receptors can, *via* the formation of death-inducing signaling complexes, trigger caspase-8 activation. This may trigger caspase-dependent apoptosis either directly *via* caspase-3 processing and/or indirectly, through BH3-only protein Bid cleavage and the mitochondria-dependent signaling (Gupta, 2003).

On the other hand, the intrinsic apoptotic pathway is triggered by all the mechanisms that we have mentioned above, including oxidative stress, mitochondria dysfunction and high levels of Ca^2+^. An important role in this type of apoptosis is played by cytochrome C which is released due to ionic imbalance, mitochondrial swelling, or the formation of a pore in the mitochondrial outer membrane (MOM; Uzdensky, 2019). The Bcl-2 family proteins are known as major inducers and regulators of the MOM pore formation. These proteins can be divided into two groups: anti-apoptotic proteins, including Bcl-2, Bcl-XL, Mcl-1 and pro-apoptotic proteins, such as Bax, Bak, and a group of BH3-only sentinels, such as Bim, Bid, p53-upregulated modulator of apoptosis (PUMA), or Bad. In cerebral ischemia, the pro-apoptotic stimuli (Bax and/or Bak) activate apoptosis by disturbing the anti-apoptotic/pro-apoptotic balance (Doyle et al., 2008). Once released from the intermembrane space, cytochrome C binds to the apoptotic protease activating factor-1 (Apaf-1), which oligomerizes and thus induces the binding and auto-activation/processing of pro-caspase 9 in a multimeric complex named apoptosome. The activated caspase-9 then activates the effector caspases 3 and 7 that subsequently cleave homeostatic-, cytoskeletal-, repair-, metabolic-, and cell signaling proteins, driving cells to apoptotic death (Niizuma et al., 2010).

In addition to cytochrome-C/caspase-3-mediated apoptosis, there is growing evidence for caspase-independent pathways in programmed cell death. This mechanism is activated *via* other pro-apoptotic proteins released from the mPTP, such as AIF or endonuclease G. AIF is not dependent on functional caspases and could, therefore, serve as an alternative death pathway after cellular energy depletion (Broughton et al., 2009).

### Glial Cells and Apoptosis

Neurons and cells of the oligodendroglial lineage are the most sensitive cellular elements and are damaged on the front line of ischemia or later they undergo delayed cell death. On the contrary, glial cells, namely microglia, and astrocytes are less vulnerable to hypoxia or hypoglycemia (Lyons and Kettenmann, 1998). The early hypotheses suggested that stroke elicits mainly the caspase-dependent apoptosis in neurons. However, the recent findings revealed that apoptosis is widespread in non-neuronal cells and that caspase-independent mechanisms play the main role in their death (Uzdensky, 2019). Such neuronal death in a caspase-independent manner was described in stroke, in which NG2 glia produce Bcl-2/E1B-19K-interacting protein 3 (BNIP3), a proapoptotic member of the Bcl-2 family proteins (Li et al., 2013). BNIP3 induces excessive mitophagy and autosis-like cell death. NG2 glia is eliminated during the early post-ischemic reperfusion, 3 h after MCAO in the adult rat brain. Compared to the other glial cell populations (astrocytes, microglia, and myelinating oligodendrocytes), the immunoreactivity of the NG2 protein-expressing glial cells, showed a rapid decrease. NG2 glia are possibly more vulnerable to severe ischemia and might be a crucial factor for axon demyelination and consequent neuronal loss (Li et al., 2013). Neuregulin-1β was proposed as a protector of NG2 glia against apoptosis, of which activity might be associated with the anti-apoptotic PI3K-Akt signaling pathway (Linying et al., 2014).

In ***astrocytic apoptosis***, it has been shown that PUMA, a BH3-only member of the Bcl-2 protein family, is upregulated during ischemia. This protein is involved in the onset and progression of several diseases and is required for p53-dependent and p53-independent forms of apoptosis. The downregulation of PUMA by siRNA transfection significantly decreases the apoptosis of astrocytes (Chen et al., 2015). PUMA could be also downregulated by the overexpression of astrocytic micro-RNA (miR-29a), thus reducing neuronal vulnerability to ischemia (Ouyang et al., 2013). On the contrary, inhibition of miR-30d increases cell autophagy but decreases cell apoptosis in astrocytes exposed to ischemia (Zhao F. et al., 2017) Interestingly, the inhibition of apoptosis in astrocytes was detected in transient receptor potential channels TRPC3/6/7 knockout mice subjected to ischemia. The deletion of these channels can reduce the nuclear factor κB (NFκB) phosphorylation (pro-apoptotic) and downregulate the expression of pro-apoptotic Bcl-2 family protein Bax (Chen et al., 2017).

The ***activated microglia*** release Toll-like receptor 4, which is a pro-inflammatory protein activating the NFκB pathway. It leads to the expression and release of the pro-inflammatory cytokines TNFα and IL-6, thus exacerbating neuronal damage and apoptosis (Zhao S. C. et al., 2017). Similarly, extracellular heat shock protein 70 (Hsp70) activates pro-inflammatory cytokines, while its intracellular localization results in a decrease in this signaling. Hsp70 is upregulated after stroke and it is not found only in microglia, but also in other cell types. Increased expression of Hsp70 causes the downregulation of dynamin, which transfers the death receptor Fas to the cell surface, inducing apoptosis, and thus Hsp70 acts neuroprotectively (Gülke et al., 2018). There are many studies regarding micro-RNA and microglia-mediated neuronal apoptosis, suggesting potential novel therapeutic interventions for ischemia. A recent study showed that miR-21 in microglia may suppress FasL production; however, in ischemic conditions, this micro-RNA is downregulated (Zhang L. et al., 2012). Another study showed that intravenously injected microglia-derived exosomes with miR-124 attenuated neuronal apoptosis after OGD (Song et al., 2019).

The caspase-dependent pathway may contribute to ***oligodendrocyte apoptosis*** in ischemia. In models of permanent FCI, oligodendrocyte injury is attenuated in cells with eliminated Bax expression or in oligodendrocytes overexpressing caspase inhibitor p35 (Shibata et al., 2000; Sánchez-Gómez et al., 2011). Oligodendrocytes could be robustly protected against ischemia-induced loss by butyrate and trichostatin A *via* histone deacetylases (HDAC) inhibition. The HDAC inhibitors downregulate caspase 3, mitigating white matter injury (Kim and Chuang, 2014). Like in previous cases of glia apoptosis, there is also evidence for the micro-RNA function in oligodendrocyte apoptosis. Here, apoptosis is suppressed through exosomal miR-134 by negatively regulating the caspase-8-dependent apoptosis pathway (Xiao et al., 2018). Excitotoxicity itself may not only act detrimentally on oligodendrocytes, but it can also cause the death of other cell types. A recent study proposed a mechanism in which oligodendrocyte injury induced by ischemia is associated with molecules released from activated microglia. According to this study, oligodendrocyte apoptosis is caused by TNFα and IL-1β, released due to the fractalkine receptor chemokine receptor 1 (CX3CR1) and its ligand (CX3CL1) axis and the p38MAPK pathway (for more detail, see Wu et al., 2016).

## Conclusion

**Figure 4:**
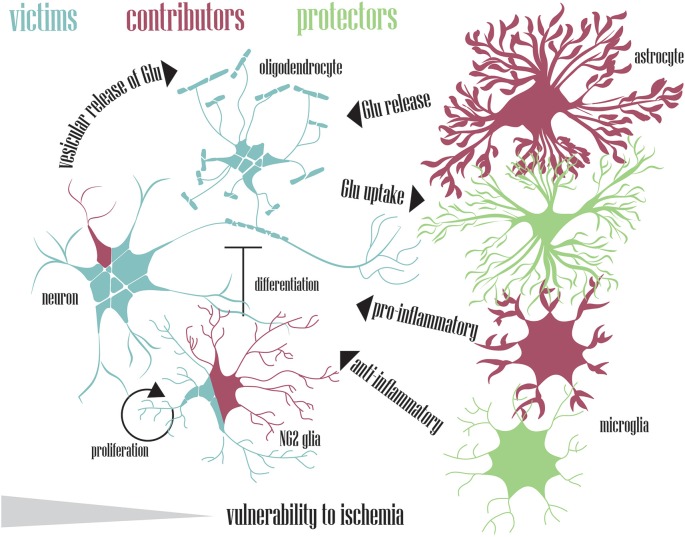
Who is the main contributor to the ischemic pathway, and who is the unsuspecting victim? The simplistic scheme shows the generalized role of individual cells and their vulnerability to ischemia.

In the present review article, we summarized available pieces of information regarding ischemia-triggered glutamate excitotoxity, its causes, and its consequences. This pathological process stems from excessive activation of glutamate receptors in neural cells and is considered a principal mechanism of neuronal death in various CNS disorders. Unfortunately, the intravenous administration of plasminogen activators remains the best and the only treatment for ischemic stroke, while timing is the key to success. The last decades have broadened our horizons, as many roles of glial cells in glutamate excitotoxicity have emerged, documenting once and for all the importance of these non-neuronal dwellers of the CNS. Recent research showed that some of them are as susceptible to overactivated glutamate signaling as neurons, some try to resist and fight against the inevitable fate. A graphical representation of such a struggle of various resident cell types is depicted in Figure 4. Addressing the question “Who is the main contributor to the ischemic pathway, and who is the unsuspecting victim?” is a heroic deed since astrocytes and microglia have both positive and negative functions in the contribution to excitotoxicity. Also, NG2 glia and neurons are not as innocent victims as they seem, because neurons participate in myelin/oligodendrocyte damage and NG2 glia lose their ability to replace dying oligodendrocytes. Therefore, all the CNS cells probably share the blame in this process. The ischemic pathway consists of many steps and seems to be so complex that targeting only its single part might not be sufficient. Nevertheless, there are more stops during the journey starting at the onset of ischemic injury, with its final destination at cell death that could serve as prospective therapeutic targets. To list some of them, the modulation of the activity of cysteine-glutamate exchangers, glutamate transporters, or apoptotic pathway appears to be one of the most promising for the development of new drugs. Additionally, a comprehensive understanding of the roles of glial cells in glutamate excitotoxicity may pave the way for new therapeutic approaches and strategies to treat many brain pathologies, such as ischemia or Alzheimer’s and Parkinson’s disease, amyotrophic lateral sclerosis or even psychiatric disorders, for example, schizophrenia.

## Author Contributions

MA, DB, JK, and JT wrote and edited the manuscript. DB worked on the figures realization. MA supervised the study.

## Conflict of Interest

The authors declare that the research was conducted in the absence of any commercial or financial relationships that could be construed as a potential conflict of interest.
